# The Hazards of Genetically Engineered Foods

**DOI:** 10.1289/ehp.114-a146

**Published:** 2006-03

**Authors:** Charles Margulis

**Affiliations:** Center for Food Safety, San Francisco, California, E-mail: cmargulis@icta.org

“Genetically Modified Foods: Breeding Uncertainty” (Schmidt 2005) overlooked many serious concerns about the environmental and health risks of this new technology. Potential problems from antibiotic-resistant genes used in gene-altered crops, risks from unintended effects of the genetic engineering process, the increases in pesticide use stemming from widespread planting of gene-spliced varieties—these and several other concerns were ignored or hardly mentioned in the lengthy article. Additional information on this topic is available from the Center for Food Safety (CFS 2000, 2004).

Instead, Schmidt’s article states that “GM agriculture is here to stay” (Schmidt 2005) and gives readers the false impression that safety and regulatory issues have been adequately addressed by industry and government. Nothing could be further from the truth. For example, regarding the risk of allergies from gene-altered foods, Schmidt stated that biotech companies avoid allergy problems by avoiding genes from the most common allergens. However, in an editorial in the *New England Journal of Medicine,* Nestle (1996) pointed out that this approach leaves many uncertainties:

Most biotechnology companies use microorganisms rather than food plants as gene donors, even though the allergenic potential of these newly introduced microbial proteins is uncertain, unpredictable, and untestable …. Because FDA requirements do not apply to foods that are rarely allergenic or to donor organisms of unknown allergenicity, the policy would appear to favor industry over consumer protection.

Schmidt (2005) went on to assert that after a 1993 study alerted them to the possibility of introducing allergens, biotech companies developed better screens and learned to abandon varieties that could not be deemed allergen-free. Far from abandoning a risky new variety 5 years after this study, industry marketed a new genetically engineered corn variety, despite warning signs that it might trigger allergies in people. Although it was registered only for nonfood uses, the altered corn, called StarLink, contaminated hundreds of food products sold in supermarkets nationwide and cost industry and farmers hundreds of millions of dollars to clean up. Aventis paid $110 million to compensate farmers for lost markets due to StarLink contamination, and analysts estimated that the company spent an additional $500 million to pay for losses to farmers, food processors, and grain handlers (Harl 2003; Jacobs 2003). Despite this and other troubling contamination episodes, such as those described by Gillis (2002), Nichols (2002), and Greenpeace (2005), the biotech industry continues to grow open fields of genetically engineered pharmaceutical crops (crops altered to produce experimental drugs or industrial proteins) that have never been assessed for their allergenic potential or other food safety issues.

Schmidt also ignored scientific concerns about the Food and Drug Administration’s (FDA) approach to gene-altered foods. Millstone et al. (1999) criticized the idea of “substantial equivalence” that the FDA uses to evaluate genetically engineered foods, calling the concept “inherently anti-scientific because it was created to provide an excuse for not requiring biochemical and toxicological tests.” In a letter published in *Nature Biotechnology*, Schenkelaars (2002) also derided the concept and noted that more appropriate testing methods would “systematically detect unintended changes in the composition of GM crops … as such changes may be of toxicological, immunological, or nutritional concern.” A lawsuit the CFS brought against the FDA exposed documents from top level scientists throughout the agency, who warned that the FDA’s equivalence-based policy was inadequate to protect against these kinds of unintended changes in gene-altered food (Alliance for Biointegrity 2004).

The purported benefits of gene-modified varieties should be examined against other agricultural approaches that have shown documented gains for food production and the environment. Schmidt (2005) cited a study of recent field trials of gene-altered rice in China that looked at a few dozen farms (Huang 2005). However, in one of the largest-ever studies of commercial rice growing, researchers found that thousands of Chinese farmers using agroecologic techniques saw yield increases of 89% while completely eliminating some of their most common pesticides (Zhu 2000). Other large-scale projects have shown that thousands of Chinese farmers using ecologic techniques significantly reduced pesticide use without expensive, patented gene-modified seeds (Yanqing 2002).

Finally, Schmidt (2005) claimed he could get no answer to his questions about industry’s plans for protecting their patented seeds in the developing world. However, that answer came in 1998, when family farm advocates exposed the biotech industry’s “terminator genes” that instill seed sterility in gene-altered varieties (Rural Advancement Foundation International 1998). This terminator technology was developed to ensure that farmers in the developing world could not reuse genetically engineered seed (ETC Group 2002). Advocates have uncovered over two dozen similar industry patents for seed sterility engineering (Rural Advancement Foundation International 1999). This technology threatens the lives of over 1.4 billion people who rely on saved seed for their daily nutritional needs, yet it is being brought to market by a genetic engineering industry that perversely promises to “feed the world.”
